# Distinct Immunophenotypes of T Cells in Bronchoalveolar Lavage Fluid From Leukemia Patients With Immune Checkpoint Inhibitors-Related Pulmonary Complications

**DOI:** 10.3389/fimmu.2020.590494

**Published:** 2021-01-21

**Authors:** Sang T. Kim, Ajay Sheshadri, Vickie Shannon, Dimitrios P. Kontoyiannis, Hagop Kantarjian, Guillermo Garcia-Manero, Farhad Ravandi, Jin S. Im, Prajwal Boddu, Lara Bashoura, Diwakar D. Balachandran, Scott E. Evans, Saadia Faiz, Wilfredo Ruiz Vazquez, Margarita Divenko, Rohit Mathur, Samantha P. Tippen, Curtis Gumbs, Sattva S. Neelapu, Aung Naing, Linghua Wang, Adi Diab, Andrew Futreal, Roza Nurieva, Naval Daver

**Affiliations:** ^1^ Department of General Internal Medicine, The University of Texas MD Anderson Cancer Center, Houston, TX, United States; ^2^ Department of Pulmonary Medicine, The University of Texas MD Anderson Cancer Center, Houston, TX, United States; ^3^ Department of Infectious Diseases, The University of Texas MD Anderson Cancer Center, Houston, TX, United States; ^4^ Department of Leukemia, The University of Texas MD Anderson Cancer Center, Houston, TX, United States; ^5^ Department of Stem Cell Transplantation and Cellular Therapy, The University of Texas MD Anderson Cancer Center, Houston, TX, United States; ^6^ Department of Immunology, The University of Texas MD Anderson Cancer Center, Houston, TX, United States; ^7^ Department of Lymphoma/Myeloma, The University of Texas MD Anderson Cancer Center, Houston, TX, United States; ^8^ Department of Genomic Medicine, The University of Texas MD Anderson Cancer Center, Houston, TX, United States; ^9^ Department of Investigational Cancer Therapeutics, The University of Texas MD Anderson Cancer Center, Houston, TX, United States; ^10^ Department of Melanoma Medical Oncology, The University of Texas MD Anderson Cancer Center, Houston, TX, United States

**Keywords:** pneumonitis, Th17/Th1 cells, checkpoint inhibitor, acute myeloid leukemia, immune-related adverse event

## Abstract

Patients with acute myeloid leukemia (AML) and myelodysplastic syndrome (MDS) treated with immune checkpoint inhibitors (ICIs) are at risk of pneumonitis as well as pneumonia (combined henceforth as ICI-related pulmonary complications). Little is known about the cellular and molecular mechanisms underlying ICI-related pulmonary complications. We characterized lymphocytes from bronchoalveolar lavage (BAL) fluid and peripheral blood from seven AML/MDS patients with pulmonary symptoms after ICI-based therapy (ICI group) and four ICI-naïve AML/MDS patients with extracellular bacterial or fungal pneumonias (controls). BAL T cells in the ICI group were clonally expanded, and BAL IFNγ^+^ IL-17^−^ CD8^+^ T and CXCR3^+^ CCR6^+^ Th17/Th1 cells were enriched in the ICI group. Our data suggest that these cells may play a critical role in the pathophysiology of ICI-related pulmonary complications. Understanding of these cell populations may also provide predictive and diagnostic biomarkers of ICI-related pulmonary complications, eventually enabling differentiation of pneumonitis from pneumonia in AML/MDS patients receiving ICI-based therapies.

## Highlights

- Th17/Th1 and IFNγ^+^ IL-17^−^ CD8^+^ T cells were enriched in bronchoalveolar lavage fluid from leukemia patients with ICI-related pulmonary complications.
- Bronchoalveolar lavage T cells were clonally expanded in patients with ICI-related complications compared with controls in terms of T cell receptor repertoire.

## Introduction

Patients with acute myeloid leukemia (AML) and myelodysplastic syndrome (MDS) are susceptible to serious infections, including pneumonia. Although immune checkpoint inhibitor (ICI)-based therapies, specifically epigenetic agent azacytidine in combination with a PD-1 inhibitor, have demonstrated encouraging responses and improved overall survival in patients with frontline or relapsed MDS or relapsed AML, ICIs are associated with immune-related adverse events, including pneumonitis (1–5). Studies have demonstrated that 10–12% of patients with a hematologic malignancy treated with ICI(s) developed pneumonitis (1, 6). Thus, AML/MDS patients receiving ICI-based therapies are at risk to develop pneumonia (due to disease and treatment-related neutropenia and immunosuppression) as well as pneumonitis (combined henceforth as ICI-related pulmonary complications). Because ICI-related pulmonary complications are life-threatening (7), understanding the pathophysiology is critical for prompt diagnosis and early intervention. Detailed characterization of the immune cells in the inflamed lung and peripheral blood (PB) from patients with AML/MDS treated with ICI-based therapies, the first step in elucidating these pathophysiologic mechanisms, would be particularly valuable. In the current study, we characterized lymphoid immune cell populations in bronchoalveolar lavage (BAL) fluid and in PB from AML/MDS patients who received ICI(s), developed pulmonary symptoms, and underwent a diagnostic bronchoscopy. As a control, we analyzed BAL fluid and PB from ICI-naïve AML/MDS patients with pulmonary symptoms who had a confirmed extracellular bacterial or fungal pneumonia.

## Materials and Methods

### Patient Selection

From March 2017 to January 2018, we reviewed for inclusion in our study 40 AML/MDS patients who underwent diagnostic bronchoscopy due to radiographic abnormalities and/or pulmonary symptoms, including fever, cough, and shortness of breath. We excluded six patients who had undergone stem cell transplantation and five patients who had received non-ICI immunotherapy. Another four patients declined to participate. Among the remaining 25 patients, 10 had received ICI therapy and 15 had not. Three of the 10 patients who had received ICI therapy were excluded; one had had pneumonia 6 weeks prior to the bronchoscopy, one had completed ICI therapy more than 12 weeks prior to the bronchoscopy, and one had lung lesions that turned out to be lymphoma. Thus, the ICI group comprised seven patients. An expert multidisciplinary committee consisting of two pulmonologists (AS and VS), one rheumatologist (SK), one infectious disease specialist (DK), and one hematologist (ND) adjudicated the presence of pneumonitis or pneumonia in these seven patients. Pneumonitis was considered the leading diagnosis if 1) radiologic patterns favored pneumonitis over pneumonia (e.g., diffuse ground-glass opacities), 2) the natural history and type of symptoms were more consistent with pneumonitis, 3) there was a clear response to corticosteroids but not antibiotics, or 4) there was histopathologic confirmation of pneumonitis or organizing pneumonia in the absence of microbiological cultures. Pneumonia was considered the leading diagnosis if 1) radiologic patterns favored pneumonia over pneumonitis (e.g., lobar consolidation), 2) the natural history and type of symptoms were more consistent with pneumonia, 3) there was a clear response to antibiotics but not corticosteroids, or 4) there was a positive microbiological culture from a lower respiratory specimen. Four patients in the ICI group met the criteria for pneumonia (hereafter, ICI-pneumonia) and three patients were determined to have pneumonitis (hereafter, ICI-pneumonitis). Two patients in the ICI-pneumonitis group had positive BAL culture results (one for *Stenotrophomonas* and one for *Enterococcus faecalis*), but the expert multidisciplinary committee determined that these were colonizations rather than active infections.

Of the 15 patients who had not received ICI therapy, eight patients were excluded because the BAL culture results were negative. Because the immune response in viral infections is distinct from that of extracellular bacterial/fungal infections, we excluded another two patients whose BAL culture results were positive for a virus. Another patient was excluded because the positive BAL culture result was clinically determined to be colonization by the expert multidisciplinary committee. The remaining four patients, whose extracellular bacterial/fungal infection was confirmed microbiologically and clinically, comprised the control group.

**Figure 1:**
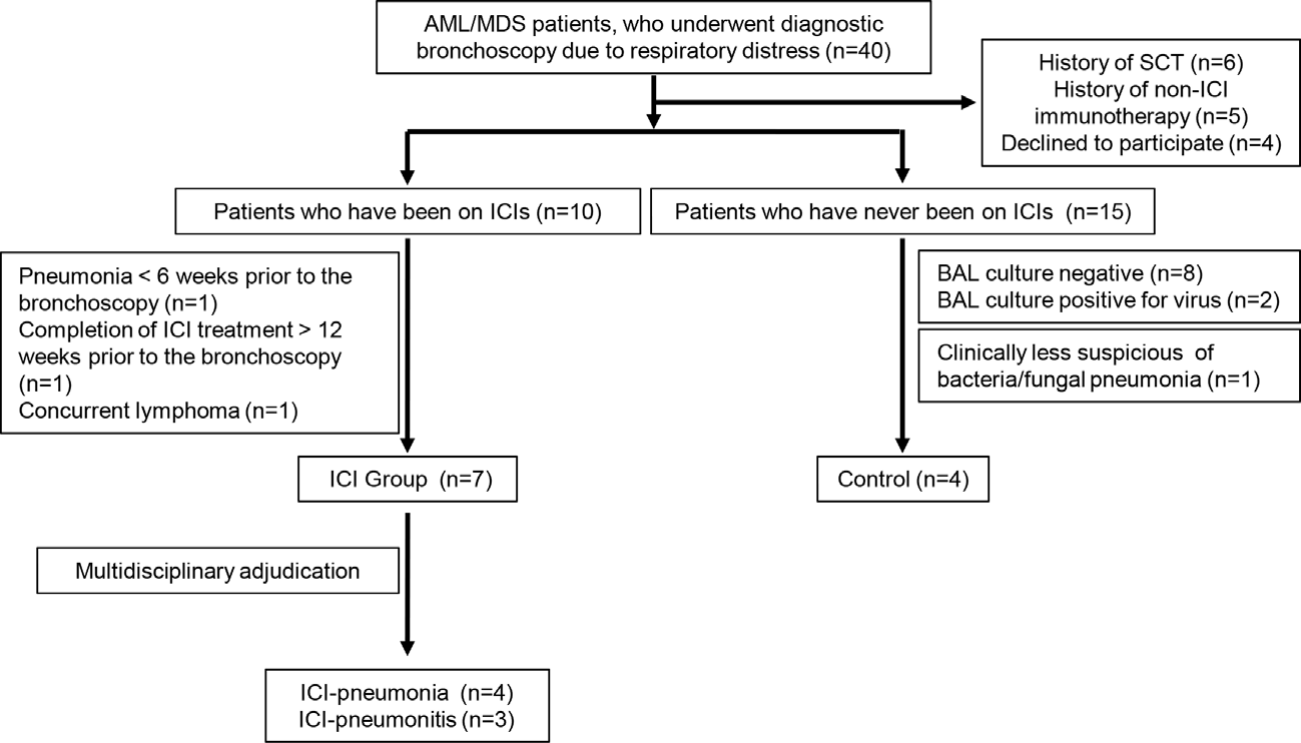
Selection process for patients in the immune checkpoint inhibitor (ICI) group and the control group. AML, acute myeloid leukemia; MDS, myelodysplastic syndrome; SCT, stem cell transplantation; BAL, bronchoalveolar lavage.

The patient selection process is summarized in **Figure 1**. Samples were collected and distributed under protocol PA15-0551 approved by the Institutional Review Board at The University of Texas MD Anderson Cancer Center and all patients provided written informed consent.

### Sample Collection

Residual BAL fluid (10–35 ml) from all participants was obtained and transported on ice to the laboratory. PB samples (15–30 ml) were collected from available patients 3 ± 3 days (mean ± SD) after the bronchoscopy. One participant in the control group (Cont_2) declined to provide a PB sample.

### Cell Isolation

After centrifugation at 1,600 rpm, the BAL fluid was stored at −80°C. BAL cells were washed with 1× phosphate buffered saline (Gibco) and cryopreserved in the presence of 90% fetal bovine serum and 10% dimethyl sulfoxide (Sigma-Aldrich). Peripheral blood mononuclear cells (PBMCs) were isolated using the Ficoll gradient technique (Sigma-Aldrich) and cryopreserved like BAL cells.

### T Cell Receptor Sequencing

DNA was extracted from cryopreserved BAL cells and PBMCs using the QIAamp DNA Mini Kit (Qiagen), and the complementarity determining region 3 of the T cell receptor beta (TCRβ) chain was amplified using the ImmunoSeq hsTCRB Kit (Adaptive Biotechnologies) and sequenced using the MiSeq platform (Illumina). Sequencing data were analyzed using the ImmunoSEQ Analyzer (Adaptive Biotechnologies), by which a series of diversity metrics were generated, including observed richness, Pielou evenness, and Simpson D (8). The clonality metric was defined as 1-Pielou evenness, where the values of clonality approach 0 when all sequences are equally abundant and perfectly even, and the values approach 1 when a single sequence makes up the entire sample.

### Flow Cytometry

Cryopreserved BAL cells and PBMCs were thawed, washed, and stained with flow cytometry antibodies to CD3, CD4, CD8, CD19, CD25, CD27, CD45A, CD56, CD127, CCR4, CCR6, CCR7, CXCR3, CXCR5, PD-1, and γδTCR. For intracellular staining, BAL cells and PBMCs were stimulated with cell activation cocktail (BioLegend) containing phorbol 12-myristate 13-acetate (PMA), ionomycin, and bredfeldin for 4 h. Cells were stained for surface molecules, fixed with BD CytoFix/CytoPerm, permeabilized with BD PERM/wash solution, and stained with antibodies to IFNγ and IL-17A. For transcription factor analysis, cells were first stained for surface molecules, then fixed and permeabilized with eBioscience^TM^ FoxP3/transcription staining buffer set. After permeabilization, cells were stained with T-bet, GATA3, RORγt, and FoxP3. Stained samples were acquired using an LSR II FORTESSA X-20 (BD Biosciences) and analyzed using FlowJo software (TreeStar). Detailed information about the flow cytometry antibodies is available in **Supplementary Table 1**.

### Identification of Immune Cell Subsets

Live immune cells were detected by gating live-dead. The lymphocytes were further examined by forward scatter and side scatter (SSC). Natural killer (NK) and NK T cells were identified by CD56 and CD3 expression. Within the CD3^+^ CD56^−^ cell population, we further gated to identify CD4^+^ T cells and CD8^+^ T cells. For CD4^+^ T cells, after gating regulatory T (Treg) cells (CD25^hi^ CD127^lo^) (9), we further divided non-Treg cells into CD45RA^+^ naïve T cells, CXCR5-expressing follicular helper T cells (Tfh) (10), and CD45RA^−^ CXCR5^−^ cells (non-Tfh effector cells). Non-Tfh effector cells were further divided into effector subsets on the basis of CXCR3, CCR4, and CCR6 expression: Th1 (CXCR3^+^ CCR6^−^), Th2 (CXCR3^−^ CCR6^−^ CCR4^+^), Th17 (CXCR3^−^ CCR6^+^), and Th17/Th1 (CXCR3^+^ CCR6^+^) cells (11–13). CD8^+^ T cells were examined by CD45RA and CCR7 staining to detect naive, central memory (Tcm), effector memory (Tem), and terminally differentiated effector memory (Tem) cells (14). Within the CD3^−^ CD56^−^ population, CD19-expressing B cells were gated. Gating strategies are shown in **Supplementary Figures 1** and **2A**. In parallel, we analyzed IFNγ- and/or IL-17A-producing T cells in BAL fluid and PB samples.

### Cytokine Measurement

IFNγ, IL-6, and IL-17A in BAL fluid were measured by multiplex ELISA, using commercially available kits (U-Plex Th17 Combo 2, Meso Scale Discovery).

### Statistical Analysis

Significant differences in means between groups were determined by the two-tailed Mann-Whitney U test, two-tailed Wilcoxon paired rank test, or one-way ANOVA. P < 0.05 was considered statistically significant. All statistical analyses were done using Prism software.

## Results

### Patient Demographic Features

The clinical characteristics of the patients are summarized in **Table 1**. Ten of the 11 patients had AML, and most (10/11) had intermediate or advanced cytogenetic characteristics. Most patients (5/7 in the ICI group; 4/4 in the control group) also had leukopenia, with a median white blood cell count of 0.4 × 10^3^/ml. Most patients in the ICI group (6/7) were receiving azacytidine in addition to the ICIs. Four patients were receiving a PD-1 inhibitor and three patients were receiving a combination of CTLA-4 and PD-1 inhibitors; two patients were receiving avelumab (3 mg/kg every 2 weeks), two patients were receiving nivolumab (3 mg/kg every 2 weeks), two patients were receiving ipilimumab (1 mg/kg every 12 weeks) plus nivolumab (3 mg/kg every 2 weeks), and one patient was receiving ipilimumab (3 mg/kg every 4 weeks) plus nivolumab (3 mg/kg every 2 weeks). All patients were on prophylactic regimen including quinolone, azol-antifungal agent, and antiviral nucleoside analogue. Patients in the ICI group developed respiratory symptoms at a median of 2.5 weeks after the initiation of ICIs; however, the range was broad (0.5 to 27.5 weeks). Three patients in the ICI group were receiving steroids at the time of bronchoscopy, at a median dose of 125 mg prednisone (or equivalent), and four patients were receiving steroids at the time of PB collection, at a median dose of 62.5 mg. Four patients in the ICI group had a positive BAL culture result, indicating extracellular bacteria with or without a virus.

**Table 1:** Basic characteristics of study patients.

Characteristic		ICI group (n=7)	ICI-pneumonia (n=4)	ICI-pneumonitis (n=3)	Controls (n=4)
Age, years, median (range)		69 (25–81)	63 (25–81)	77 (52–79)	62.5 (55–79)
Sex (male/female)		2/5	0/4	2/1	3/1
Primary tumor					
AML		6	3	3	4
MDS		1	1	0	0
ECOG performance status, median (range)		1.5 (1–2)	1.5 (1–2)	1.5 (1–2)	1.5 (1–2)
Patients with antecedent hematologic disorder		1	1	0	2
Cytogenetic group	Adverse	5	4	1	1
	Intermediate	2	0	2	2
	Favorable	0	0	0	1
Molecular mutations (minimum ≥ 2 cases)	TP53	3	2	1	0
	FLT3	2	1	1	0
	DNMT3A	0	0	0	2
Peripheral blood WBC count at bronchoscopy, ×10^3^/ml, median (range)		0.4 (0.2–17.5)	0.5 (0.2–6.2)	0.4 (0.4–17.5)	0.4 (0.1–3.2)
Peripheral blood blasts at bronchoscopy, %, median (range)		25 (0–68) (n=5)	16 (7–25) (n=2)	40 (0–68) (n=3)	2.5 (0–5) (n=2)
BM blasts on most recent BM biopsy prior to bronchoscopy, %, median (range)		44 (10–90)	23.5 (10–90)	58 (44–84)	34 (1–87)
ICI treatment status (frontline/salvage)		3/4	3/1	0/3	n/a
Treatment regimen					
Azacytidine + ICI-based		6	3	3	0
Non-azacytidine + ICI-based		1	1	0	0
Fludarabine + cytarabine + idarubicin + sorafenib		0	0	0	1
Cytarabine + idarubicin		0	0	0	1
Non-immune investigational small molecule(s)		0	0	0	2
Best response to treatment regimen (CR or CRp)		1	0	1	1
Patients actively on ICI treatment at bronchoscopy		7	4	3	n/a
Discontinuation of ICI protocol prior to bronchoscopy		0	0	0	n/a
ICI regimen					
PD-1 inhibitor		4	2	2	n/a
CTLA-4 inhibitor		0	0	0	n/a
Combined PD-1 and CTLA-4 inhibitors		3	2	1	n/a
Admission status at bronchoscopy, routine floor/ICU		7	4/0	3/0	4/0
Patients receiving steroid at time of bronchoscopy					
Dose of prednisone (or equivalent) at time of bronchoscopy, mg, median (range)		125 (50–300) (n=3)	50 (n=1)	212.5 (125–300) (n=2)	n/a (n=0)
Patients receiving steroid at time of blood draw					
Dose of prednisone (or equivalent) at time of blood draw, mg, median (range)		62.5 (30,150) (n=4)	35 (20–50) (n=2)	112.5 (75–150) (n=2)	n/a (n=0)
Duration, weeks, median (range)					
From first ICI infusion to respiratory symptoms		2.5 (0.5–27.5)	1.5 (0.5–3.5)	3.5 (0.5–27.5)	n/a
From first ICI infusion to bronchoscopy		4 (0.5–28)	3.5 (0.5–6.5)	4 (2–28)	n/a
From last ICI infusion to respiratory symptoms		0.5 (0.5–2)	0.5 (0.5–1.5)	2 (0.5–2)	n/a
From last ICI infusion to bronchoscopy		2 (0.5–5.5)	1.5 (0.5–5.5)	2 (1.5–2)	n/a
Patients receiving prophylactic antibiotic at time of bronchoscopy		7	4	3	4
Antibacterial agent					
Levofloxacin		7	4	3	2
Ciprofloxacin		0	0	0	2
Antifungal agent					
Fluconazole		2	1	1	2
Voriconazole		3	2	1	0
Posaconazole		1	1	0	1
Isavuconazole		1	0	1	0
Esavuconazole		0	0	0	1
Antiviral agent					
Valaciclovir		6	3	3	4
Acyclovir		1	1	0	0
Patients admitted ≤6 weeks prior to bronchoscopy		3	2	1	2
BAL fluid culture results					
Negative		3	2	1	0
Virus		0	0	0	0
Extracellular bacteria		3	1	2	2
Fungi		0	0	0	2
Extracellular bacteria and virus		1	1	0	0
Findings on chest CT					
Infectious pneumonia		4	4	0	4
Hypersensitivity pneumonitis		2	0	2	0
Organizing pneumonia		1	0	1	0

ICI, immune checkpoint inhibitor; AML, acute myeloid leukemia; MDS, myelodysplastic syndrome; ECOG, Eastern Cooperative Oncology Group; WBC, white blood cells; BM, bone marrow; n/a, not applicable; CR, complete remission; CRp, complete remission without platelet recovery; ICU, intensive care unit; BAL, bronchoalveolar lavage; CT, computed tomography.

### Manual Differentials of Bronchoalveolar Lavage and Peripheral Blood

**Figure 2:**
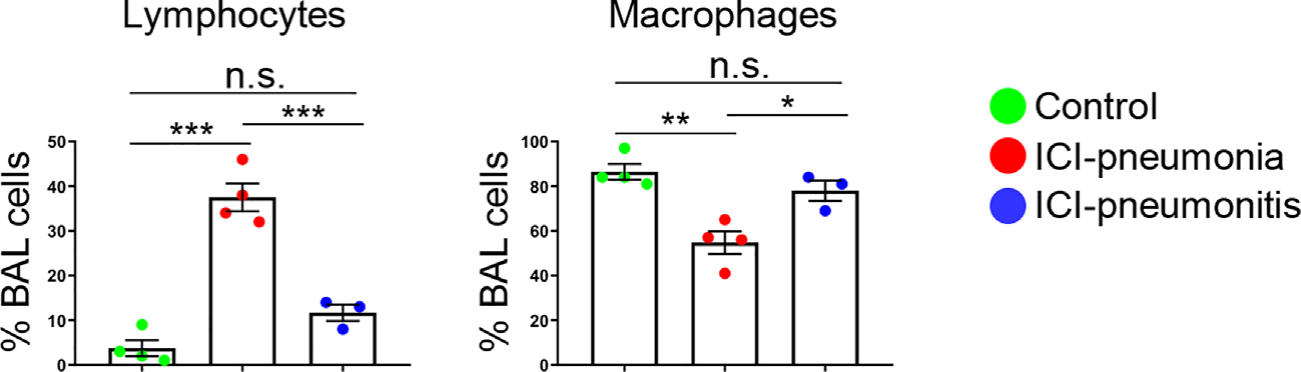
Proportion of major bronchoalveolar lavage (BAL) cell subsets with manual differential tests. Bars indicate the mean and the SEM. One-way ANOVA. *P < 0.05, **P < 0.01, ***P < 0.001, n.s., not significant. ICI, immune checkpoint inhibitor.

For all patients, manual leukocyte differentials of BAL and PB cells were counted as standard of care (**Table 2**). The differentiation tests of PB from four patients, two in the ICI group and two in the control group, could not be performed owing to severe leukopenia. The frequency of BAL lymphocytes was significantly higher in the ICI group than in the control group (mean ± SD; ICI *vs*. control; 26.4 ± 15.0 *vs*. 3.8 ± 3.6; P = 0.01), whereas the mean frequency of BAL macrophages was significantly lower in the ICI group than in the control group (mean ± SD; ICI *vs*. control; 64.7 ± 15.0 *vs*. 86.5 ± 7.1; P = 0.03). This trend was more prominent in the ICI-pneumonia group than in the ICI-pneumonitis group (**Figure 2**). Consistently, the mean frequency of PB lymphocytes in the ICI group was higher than in the control group; however, the differences did not reach statistical significance (mean ± SD; ICI *vs*. control; 44.8 ± 15.0 *vs*. 19.0± 11.3; P = 0.09).

**Table 2:** Manual differentiations of bronchoalveolar lavage (BAL) and peripheral blood (PB) samples.

BAL	ICI group (n=7)	ICI-pneumonia (n=4)	ICI-pneumonitis (n=3)	Control (n=4)
Cell subsets, %, median (range)				
Lymphocyte	32 (8–46)	36 (32–46)	13 (8–14)	2.5 (1–9)
Macrophage	65 (41–84)	56.5 (41–65)	81 (69–84)	84 (81–97)
Neutrophil	0 (0–1)	0.5 (0–1)	0 (0–0)	0 (0–0)
Eosinophil	0 (0–0)	0 (0–0)	0 (0–0)	0 (0–0)
Basophil	0 (0–0)	0 (0–0)	0 (0–0)	0 (0–0)
Others	9 (1–17)	7 (1–12)	11 (2–17)	11.5 (1–13)
PB*	**ICI group (n=5)**	**ICI-pneumonia (n=2)**	**ICI-pneumonitis (n=3)**	**Control (n=2)**
Cell subsets, %, median (range)				
Lymphocyte	39 (32–67)	46 (39–53)	33 (32–67)	19 (11–27)
Macrophage	5 (0–20)	7 (1–13)	5 (0–20)	47.5 (12–83)
Neutrophil	16 (0–33)	25.5 (18–33)	13 (0–16)	28 (0–56)
Eosinophil	1 (0–1)	1 (1–1)	0 (0–1)	2.5 (0–5)
Basophil	1 (0–4)	1 (1–1)	0 (0–4)	0 (0–0)
Others^†^	28 (0–68)	19.5 (11–28)	44 (0–68)	3 (0–6)

ICI, immune checkpoint inhibitor; BAL, bronchoalveolar lavage; PB, peripheral blood.

*PB differentiation tests could not be performed in two patients in the ICI-pneumonia group and two patients in the control group owing to severe leukopenia.

^†^Blasts are included.

### Distinct Immune Landscape of Bronchoalveolar Lavage T Cells in the Immune Checkpoint Inhibitor Group

**Figure 3:**
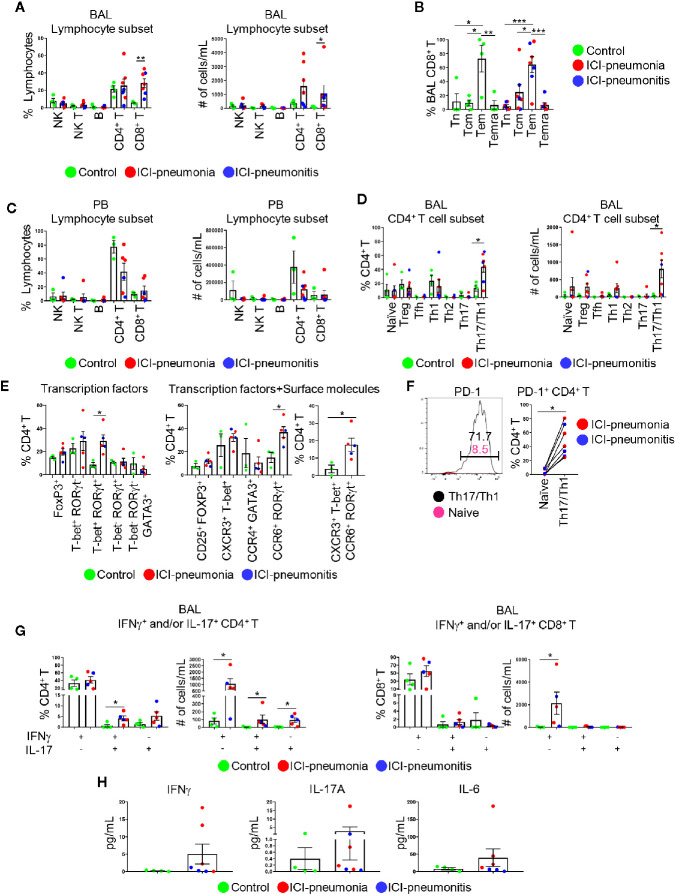
Characterization of lymphoid immune cell subsets in bronchoalveolar lavage (BAL) fluid and peripheral blood (PB). **(A)** Proportions of major BAL immune cell subsets within live lymphocytes and absolute cell numbers in 1 ml BAL fluid. NK, natural killer cells; NK T, natural killer T cells; B, B cells. Bars indicate the mean and the SEM. Mann-Whitney U test. *P<0.05, **P<0.01. **(B)** Proportions of CD8^+^ T cell subsets within BAL CD8^+^ T cells. Tn, naïve T cells; Tcm, central memory T cells; Tem, effector memory T cells; Temra, terminally differentiated T cells. Bars indicate the mean and the SEM. One-way ANOVA. *P<0.05, **P<0.01, ***P<0.001. **(C)** Proportions of major PB immune cell subsets within live lymphocytes and absolute cell numbers in 1 ml PB. Bars indicate the mean and the SEM. **(D)** Proportions of CD4^+^ T cell subsets within CD4^+^ T cells and absolute cell numbers in 1 ml BAL fluid. Treg, regulatory T cells; Tfh, follicular helper T cells. Bars indicate the mean and the SEM. Mann-Whitney U test. *P<0.05. **(E)** Proportion of BAL CD4^+^ T cells expressing indicated transcription factors (left), transcription factors and surface molecules (middle and right). Bars indicate the mean and the SEM. Mann-Whitney U test. *P<0.05. **(F)** PD-1 on BAL naïve CD4^+^ T cells and BAL CXCR3^+^ CCR6^+^ Th17/Th1 cells. Left panel shows one of the most representative plots and right panel shows quantification. Wilcoxon paired rank test. *P<0.05. **(G)** Proportions and absolute numbers of IFNγ- and/or IL-17-producing CD4^+^ and CD8^+^ T cells in BAL fluid. Bars indicate the mean and the SEM. Mann-Whitney U test. *P<0.05. **(H)** Levels of IFNγ, IL-6, and IL-17A in BAL fluid measured by multiplex ELISA. Bars indicate the mean and the SEM.

Given the enrichment of lymphocytes in BAL fluid and PB, we focused on characterizing lymphocytes and enumerating major lymphocytic subsets in both BAL fluid and PB (**Figure 3**; **Supplementary Table 2**). The absolute number of lymphocytes per 1 ml BAL fluid was higher in the ICI group than in the control group (mean ± SD; ICI *vs*. control: 23,791 ± 41,142 cells *vs*. 1,285 ± 1,051 cells; P = 0.01; **Supplementary Table 2**). The proportions of NK cells (CD3^−^ CD56^+^), NK T cells (CD3^+^ CD56^+^), B cells (CD3^−^ CD56^−^ CD19^+^), and CD4^+^ T cells (CD3^+^ CD4^+^ CD8^−^) were similar between the ICI and control groups (**Figure 3A**; **Supplementary Figure 1**). BAL CD8^+^ T cells (CD3^+^ CD4^−^ CD8^+^) were significantly expanded in the ICI group compared with the control group in terms of frequencies and numbers (frequency: mean ± SD; ICI *vs*. control; 28.4 ± 13.0% *vs*. 5.8 ± 1.2%; P = 0.006) (absolute cell numbers: mean ± SD; ICI *vs*. control; 28.4 ± 13.0% *vs*. 5.8 ± 1.2%). Most of these CD8^+^ T cells were CD45RA^−^ CCR7^−^ effector memory cells (64.2 ± 30.7%; **Figure 3B**), suggesting that these cells play a role in ICI-related pulmonary complications. The frequencies and absolute number of cells for lymphocytic immune subsets in the PB samples were similar between the ICI and control groups (**Figure 3C**).

Next, we delineated CD4^+^ T cell subsets on the basis of chemokine/cytokine receptor expression, including regulatory T cells, naïve T cells, follicular helper T cells, Th1, Th2, Th17, and Th17/Th1 cells (9–13) (**Figure 3D**; **Supplementary Figure 2**). Although the proportions of PB CD4^+^ T cell subsets were similar between the ICI and control groups (**Supplementary Figure 3**), BAL Th17/Th1 cells were significantly expanded in the ICI group compared with the control group (mean ± SD; ICI *vs*. control; 43.8 ± 20.5% *vs*. 13.3 ± 8.8%; P = 0.04; **Figure 3D**). For selected patients (n=3 in control; n=3 in ICI-pneumonia; n=2 in ICI-pneumonitis), along with chemokine/cytokine receptors, we also investigated expression of key transcription factors including T-bet (Th1), GATA3 (Th2), RoRγT (Th17), and FoxP3 (Treg) (**Figure 3E**; **Supplementary Figure 3**) (10). Consistent with data in **Figure 3D**, we observed enrichment of T-bet^+^ RORγt^+^ (Th1) and CXCR3^+^ T-bet^+^ CCR6^+^ RORγt^+^ (Th1/Th17) cells in BAL CD4^+^ T cells in the ICI group. Most (48.0 ± 22.5%) BAL Th17/Th1 cells expressed PD-1 (**Figure 3F**), suggesting that these cells had persistent antigen exposure (15).

To evaluate the functionality of the T cells, we performed intracellular staining to assess IFNγ- and/or IL-17-producing T cells (**Figure 3G**; **Supplementary Figure 4**). In BAL fluid, the absolute number of IFNγ- and/or IL-17-producing CD4^+^ T cells was higher in the ICI group than in the control group. In addition to the number of cells, the frequency of IFNγ^+^ IL-17^+^ CD4^+^ T cells in BAL fluid was significantly higher in the ICI group than in the control group (mean ± SD; ICI *vs*. control; 4.1 ± 2.4% *vs*. 0.7 ± 1.3%; P = 0.03; **Figure 3G**). Consistently, although proportions of IFNγ^+^ IL-17^−^ CD8^+^ cells in BAL fluid were similar between the two groups, the absolute number of these cells was higher in the ICI group than in the control group (mean ± SD; ICI *vs*. control; 2135.0 ± 2203.0 cells *vs*. 30.0 ± 44.0 cells; P = 0.01; **Figure 3G**). Although not statistically significant, the levels of soluble IFNγ, as well as IL-6 and IL-17A, key cytokines for Th17 cell differentiation, plasticity, and function (10, 16), in the BAL fluid were higher in the ICI group than in the control group (**Figure 3H**). IFNγ- and/or IL-17-producing CD4^+^ and CD8^+^ T cells in PB were comparable between the ICI and control groups (**Supplementary Figure 4**).

### Clonally Expanded Bronchoalveolar Lavage T Cells in the Immune Checkpoint Inhibitor Group

**Figure 4:**
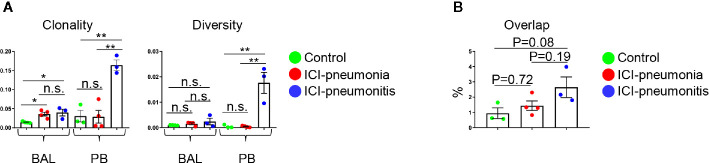
**(A)** Clonality and diversity of T cells in the bronchoalveolar lavage (BAL) fluid and peripheral blood (PB). Bars indicate the mean and the SEM. One-way ANOVA. *P<0.05, **P<0.01, n.s., not significant. **(B)** Quantification of overlapped T-cell receptor sequences between BAL and PB. Bars indicate the mean and the SEM. One-way ANOVA.

We analyzed the TCR repertoire in 11 matched BAL fluid and PB samples (**Figure 4**). T cells in the ICI group, especially the BAL T cells, were significantly clonally expanded compared with the control group (mean ± SD; ICI *vs*. control; 0.077 ± 0.011 *vs*. 0.014 ± 0.002; P = 0.006). Clonality and diversity of PB T cells was higher in the ICI-pneumonitis group than in the ICI-pneumonia and control groups (clonality: mean ± SD; ICI-pneumonitis *vs*. ICI-pneumonia *vs*. control; 0.16 ± 0.02 *vs*. 0.03 ± 0.03 *vs*. 0.03 ± 0.03, P = 0.001) (diversity: mean ± SD; ICI-pneumonitis *vs*. ICI-pneumonia *vs*. control; 0.02 ± 0.01 *vs*. 0.0004 ± 0.0003 *vs*. 0.0004 ± 0.0006; P = 0.001) (**Figure 4A**). We investigated the overlapped T cell clones in BAL and PB (**Figure 4B**; **Supplementary Figure 6**). Although not reached statistical significance, a greater degree of overlap was observed in the ICI-pneumonitis, compared with ICI-pneumonia and controls (**Figure 4B**), suggesting that ICI-pneumonitis might be a systemic inflammation.

Subgroup analysis of the ICI group based on ICI regimen [PD-1 inhibitor (n=4) compared with combined CTLA-4 and PD-1 inhibitors (n=3)] and concurrent steroid treatment at the time of biospecimen collection [steroid (n=3) compared with no steroid (n=4)] revealed no differences in immunophenotypes or TCR repertoire (data not shown).

## Discussion

AML/MDS patients receiving ICIs can develop pneumonia due to their diseases and leukopenia and pneumonitis as an immune-related adverse event. As the first step to investigate mechanisms underlying these ICI-related pulmonary complications, we immunoprofiled BAL fluid and PB samples from AML/MDS patients with pulmonary complications after ICI therapy. Compared with control patients (ICI-naïve AML/MDS patients with bacterial/fungal pneumonia), patients with ICI-related pulmonary complications had enriched lymphocytes, especially Th17/Th1 cells and IFNγ^+^ CD8^+^ T cells, in BAL fluid, as well as clonally expanded BAL T cells. Subgroup analysis of the ICI group revealed that patients with ICI-pneumonia had predominant BAL lymphocytes and patients with ICI-pneumonitis had enhanced T cell clonality and diversity in PB. Combined, our data suggest that distinct T cell responses occur in patients with ICI-related pulmonary complications.

Th17 cells are highly plastic and can be differentiated into CXCR3^+^ CCR6^+^ IFNγ^+^ IL-17^+^ Th17/Th1 cells. Studies have shown that Th17/Th1 cells play an important role in the pathogenesis of autoimmune diseases (17). Indeed, Th17/Th1 cells were shown to be enriched in inflammatory sites of autoimmune diseases including the colon in Crohn’s disease, cerebrospinal fluid in multiple sclerosis, and synovial fluid in rheumatoid arthritis and juvenile idiopathic arthritis (13, 18–21). Recent studies revealed that these cells are also enriched in the BAL fluid from patients with sarcoidosis (22–24). Based on the studies, we speculate that enrichment of BAL Th17/Th1 cells in our study is not a non-specific finding secondary from inflammation; rather we hypothesize that these BAL Th17/Th1 cells play a key role in the pathogenesis of ICI-related pulmonary complications. Our hypothesis is partially supported by the *in vivo* and *in vitro* observations that genetic or pharmacologic depletion of PD-1 enhanced Th17 responses in a mouse model of allergic asthma (25). Further studies are warranted to investigate the generation and function of Th17/Th1 cells in ICI-related pulmonary complications.

About 3–5% of patients with solid tumors develop pneumonitis after ICI therapy (6). The pneumonitis with solid tumors is one of early immune-related adverse events with onset at a median of 2.8 months, with a wide range (9 days to 19.2 months) (6). Suresh *et al*. recently characterized BAL fluid from patients with solid tumors who developed ICI-induced pneumonitis, and that analysis revealed prominent lymphocytes, especially IFNγ^+^ CD8^+^ T cells (26). Patients in our cohort also developed respiratory symptoms early after the initiation of ICIs (median: 2.5 weeks), and BAL analyses revealed prominent lymphocytes in the ICI group (**Table 2**). Importantly, we observed enrichment of IFNγ^+^ CD8^+^ T cells, but we also observed enrichment of Th17/Th1 cells, suggesting that there are shared and distinct mechanisms underlying ICI-induced pneumonitis depending on the tumor type. Dissection of immune profiles of ICI-induced pneumonitis between patients with solid tumors and those with leukemia would be of future interest.

The difference in immunophenotypes between ICI-pneumonia and ICI-pneumonitis is unclear. Although the difference was not statistically significant, we found that the median onset of respiratory symptoms was shorter in the ICI-pneumonia group than in the ICI-pneumonitis group (ICI-pneumonia *vs*. ICI-pneumonitis; 1.5 *vs*. 3.5 weeks), and proportions of BAL lymphocytes were higher in the ICI-pneumonia group, suggesting that BAL lymphocytes, most likely T cells, of the ICI-pneumonia actively proliferate and/or survive longer compared with BAL T cells of the ICI-pneumonitis. Considering these findings, we speculate that patients with ICI-pneumonia might have more enhanced T cell memory responses than patients with ICI-pneumonitis. Not mutually exclusive, it is also possible that antigens in ICI-pneumonia have heightened antigenicity compared with those in ICI-pneumonitis. In contrast, we found that clonality and diversity of circulating T cells were higher in the ICI-pneumonitis group than in the ICI-pneumonia group. Collectively, we hypothesis that exogenous antigens (bacteria and/or fungus) in the ICI-pneumonia might provide strong TCR and toll-like receptor signal, which induce global and indirect T cell activation/reactivation with prolonged T cell survival. In contrast, endogenous antigens (self-antigens or tumor antigens) might specifically activate T cells recognizing these endogenous antigens, resulting in enhanced TCR clonality. Our hypothesis is supported by the study, showing enhanced TCR clonality in inflamed joints (synovial fluid) and blood of patients with psoriatic arthritis, one of the most common autoimmune diseases (27). In addition, previous studies showed an increase of clonality and diversity of T cells in patients with immune-related adverse events (28–31). However, given our small sample size and the unstable PB cells in AML/MDS, we could not make any conclusions at present. Future studies investigating cell proliferation (Ki67), apoptosis (annexin V, DAPI), exhaustion (LAG3, TIM3, PD-1, TIGIT), and anti-apoptosis gene expression (Bcl2, Bcl-xL) in BAL/PB cells between ICI-pneumonia and ICI-pneumonitis will enable us to dissect mechanisms of ICI-pneumonia and ICI-pneumonitis. Nevertheless, BAL differentiation counts and/or TCR repertoires in PB might be a potential biomarker to differentiate ICI-pneumonia from ICI-pneumonitis.

Our study has a few limitations. First, because of the small number of patients analyzed, these data are inconclusive. Second, this study does not have a control group comprising patients with solid tumors who developed ICI-induced pneumonitis. In addition, some patients in the ICI group were on azacitidine in addition to ICI and azacitidine can alter immune profiles (32, 33). Indeed, studies revealed increased numbers of Tregs and decreased numbers of CD8^+^ T cells and Th1 cells after azacitidine therapy (32, 33). Our study showed enrichment of BAL Th17/Th1 cells and IFNγ^+^ IL-17^−^ CD8^+^ T cells in the ICI group while studies showed that stable and decreased numbers of Th17 and CD8^+^ T cells with azacitidine. Together, we speculated that azacitidine might not have influenced our main observations; however, given that epigenetic mechanisms are critical in regulating T cell lineage commitment (34), ICI-naïve AML/MDS patients with azacitidine monotherapy should also be served as a control group. Third, three participants in the ICI group were receiving steroids at the time of BAL fluid collection and four at the time of PB sample collection, which might have altered the immune profiles.

In this study, the samples were mainly obtained from the pilot phase IB trials initiated in 2017-2018 at the University of Texas MD Anderson Cancer Center, comparing efficacy and safety of ICI-based therapies in patients with AML/MDS. With the initial encouraging results, we have recently opened a number of additional ICI-based trials for AML and MDS including clinical trials of azacitidine + nivolumab + ipilimumab (NCT02397720), azacitidine+venetoclax+nivolumab (NCT02397720), azacitidine + venetoclax +avelumab (NCT03390296), azacitidine + venetoclax + TIM3 antibody (NCT04150029), with larger numbers of participants (150–180) expected to be enrolled at the MD Anderson across these phase IB/II larger trials. In this manuscript, we aimed to generate hypothesis rather test the hypothesis. Since 10–12% of the AML/MDS patients develops pneumonitis (1, 6), from these upcoming trials, we expect to collect 15–22 BAL and matching PB samples from AML/MDS patients with ICI-pneumonitis (and similar numbers of the samples from AML/MDS patients with ICI-pneumonia as well). Detailed investigation of cell survivals, proliferation, and exhaustion are warranted in future studies to dissect underlying mechanisms between ICI-pneumonia and ICI-pneumonitis. Based on distinct TCR repertoires between ICI-pneumonia and ICI-pneumonitis, analysis of both TCR α and β chains are also needed in the future studies. ICI-naïve AML/MDS patients who develops pulmonary complications after azacitidine monotherapy will be served as a control group in future studies. Additionally, the standard therapy for frontline older AML has now transitioned to azacitidine+venetoclax, and it is possible this will emerge as a more effective therapy in frontline MDS as well. We have a large number of patients treated with azacitidine and venetoclax for both AML and MDS and plan to assess BAL samples on these patients as well to serve as an additional future control. Finally, although we did not see differences of immune profiles of concurrent steroid treatment, the analysis might be underpowered. Future studies should carefully model the use of steroids and standardize BAL collection before steroids are administered. In some cases of life-threatening pneumonitis, steroid therapy is empirically initiated prior to the diagnostic bronchoscopy. Nevertheless, larger numbers of the samples in the future studies will enable us to perform subgroup analysis (steroid *vs*. no steroid) with adequate power. In conclusion, our study showed distinct immunophenotypes of T cells in BAL fluid in AML/MDS patients with ICI-related pulmonary complications. Detailed molecular and cellular characterization of immune cells in a larger number of patients, with appropriate controls, may provide insights into the mechanisms of pneumonitis in AML/MDS treated with ICIs-based therapy, as well as provide diagnostic biomarkers to differentiate pneumonitis from pneumonia and potentially predict the severity of the pneumonitis.

## Data Availability Statement

The raw data supporting the conclusions of this article will be made available by the authors, without undue reservation.

## Ethics Statement

The studies involving human participants were reviewed and approved by The IRB at the University of Texas MD Anderson Cancer Center. The patients/participants provided their written informed consent to participate in this study.

## Author Contributions

SK performed experiments, analyzed the data, and wrote the manuscript. AS, VS, HK, GG-M, FR, LB, DB, SE, SF, and ND provided samples. JI, WR-V, MD, SN, RM, LW, ST, CG, and AF performed experiments and discussed results. AD and PB analyzed the data and discussed results. AN discussed the results. SK, AS, VS, DK, and ND were responsible for adjudication of the patients. RN and ND oversaw the study and discussed results. All authors reviewed and edited the manuscript. All authors contributed to the article and approved the submitted version.

## Funding

This work was supported by The University of Texas MD Anderson Cancer Center Division of Internal Medicine Developmental Funds (SK), the NIH RO1 grants (RN: R01HL141966 and R01HL143520) and CPRIT grant (RN: RP190326).

## Conflict of Interest

SN has received research support from Kite/Gilead, Cellectis, Poseida, Merck, Acerta, Karus, BMS, Unum Therapeutics, Allogene, and Precision Biosciences; served as consultant and advisory board member for Kite/Gilead, Celgene, Novartis, Unum Therapeutics, Pfizer, Merck, Precision Biosciences, Cell Medica, Incyte, Allogene, Calibr, and Legend Biotech; and has patents related to cell therapy. ND has received research funding from Daiichi Sankyo, Bristol-Myers Squibb, Pfizer, Karyopharm, Sevier, Genentech, and ImmunoGen and has served in a consulting or advisory role for Daiichi Sankyo, Bristol-Myers Squibb, Pfizer, Novartis, Celgene, AbbVie, and Agios.

The remaining authors declare that the research was conducted in the absence of any commercial or financial relationships that could be construed as a potential conflict of interest.

## References

[1] Daver N Basu S Garcia-Manero G Cortes J Ravandi F Jabbour E Phase IB/II study of nivolumab in combination with azacytidine in patients with relapsed acute myeloid leukemia. Madrid, Spain: EHA Learning Center (2017). p. S474.

[2] Alatrash G Daver N Mittendorf EA Targeting Immune Checkpoints in Hematologic Malignancies. Pharmacol Rev (2016) 68(4):1014–25. 10.1124/pr.116.012682 PMC11060433 27664133

[3] Boddu P Kantarjian H Garcia-Manero G Allison J Sharma P Daver N The emerging role of immune checkpoint based approaches in AML and MDS. Leukemia lymphoma (2018) 59(4):790–802. 10.1080/10428194.2017.1344905 28679300 PMC5872841

[4] Larkin J Chiarion-Sileni V Gonzalez R Grob JJ Cowey CL Lao CD Combined Nivolumab and Ipilimumab or Monotherapy in Untreated Melanoma. N Engl J Med (2015) 373(1):23–34. 10.1056/NEJMoa1504030 26027431 PMC5698905

[5] Postow MA Sidlow R Hellmann MD Immune-Related Adverse Events Associated with Immune Checkpoint Blockade. N Engl J Med (2018) 378(2):158–68. 10.1056/NEJMra1703481 29320654

[6] Naidoo J Wang X Woo KM Iyriboz T Halpenny D Cunningham J Pneumonitis in Patients Treated With Anti-Programmed Death-1/Programmed Death Ligand 1 Therapy. J Clin Oncol (2017) 35(7):709–17. 10.1200/JCO.2016.68.2005 PMC5559901 27646942

[7] Nishino M Sholl LM Hodi FS Hatabu H Ramaiya NH Anti-PD-1-Related Pneumonitis during Cancer Immunotherapy. N Engl J Med (2015) 373(3):288–90. 10.1056/NEJMc1505197 PMC4539956 26176400

[8] Reuben A Gittelman R Gao J Zhang J Yusko EC Wu CJ TCR Repertoire Intratumor Heterogeneity in Localized Lung Adenocarcinomas: An Association with Predicted Neoantigen Heterogeneity and Postsurgical Recurrence. Cancer Discov (2017) 7(10):1088–97. 10.1158/2159-8290.CD-17-0256 PMC5628137 28733428

[9] Yu N Li X Song W Li D Yu D Zeng X CD4(+)CD25 (+)CD127 (low/-) T cells: a more specific Treg population in human peripheral blood. Inflammation (2012) 35(6):1773–80. 10.1007/s10753-012-9496-8 22752562

[10] Craft JE Follicular helper T cells in immunity and systemic autoimmunity. Nat Rev Rheumatol (2012) 8(6):337–47. 10.1038/nrrheum.2012.58 PMC3604997 22549246

[11] Acosta-Rodriguez EV Rivino L Geginat J Jarrossay D Gattorno M Lanzavecchia A Surface phenotype and antigenic specificity of human interleukin 17-producing T helper memory cells. Nat Immunol (2007) 8(6):639–46. 10.1038/ni1467 17486092

[12] Becattini S Latorre D Mele F Foglierini M De Gregorio C Cassotta A T cell immunity. Functional heterogeneity of human memory CD4(+) T cell clones primed by pathogens or vaccines. Science (6220) 2015) 347:400–6. 10.1126/science.1260668 25477212

[13] van Langelaar J van der Vuurst de Vries RM Janssen M Wierenga-Wolf AF Spilt IM Siepman TA T helper 17.1 cells associate with multiple sclerosis disease activity: perspectives for early intervention. Brain J Neurol (2018) 141(5):1334–49. 10.1093/brain/awy069 29659729

[14] Sallusto F Geginat J Lanzavecchia A Central memory and effector memory T cell subsets: function, generation, and maintenance. Annu Rev Immunol (2004) 22:745–63. 10.1146/annurev.immunol.22.012703.104702 15032595

[15] Rao M Valentini D Dodoo E Zumla A Maeurer M Anti-PD-1/PD-L1 therapy for infectious diseases: learning from the cancer paradigm. Int J Infect Dis IJID (2017) 56:221–8. 10.1016/j.ijid.2017.01.028 28163164

[16] Harbour SN DiToro DF Witte SJ Zindl CL Gao M Schoeb TR TH17 cells require ongoing classic IL-6 receptor signaling to retain transcriptional and functional identity. Sci Immunol (2020) 5(49):eaaw2262. 10.1126/sciimmunol.aaw2262 32680955 PMC7843024

[17] Kamali AN Noorbakhsh SM Hamedifar H Jadidi-Niaragh F Yazdani R Bautista JM A role for Th1-like Th17 cells in the pathogenesis of inflammatory and autoimmune disorders. Mol Immunol (2019) 105:107–15. 10.1016/j.molimm.2018.11.015 30502718

[18] Globig AM Hennecke N Martin B Seidl M Ruf G Hasselblatt P Comprehensive intestinal T helper cell profiling reveals specific accumulation of IFN-gamma+IL-17+coproducing CD4+ T cells in active inflammatory bowel disease. Inflammation Bowel Dis (2014) 20(12):2321–9. 10.1097/MIB.0000000000000210 25248005

[19] Maggi L Mazzoni A Cimaz R Liotta F Annunziato F Cosmi L Th17 and Th1 Lymphocytes in Oligoarticular Juvenile Idiopathic Arthritis. Front Immunol (2019) 10:450. 10.3389/fimmu.2019.00450 30930898 PMC6428030

[20] Kotake S Yago T Kobashigawa T Nanke Y The Plasticity of Th17 Cells in the Pathogenesis of Rheumatoid Arthritis. J Clin Med (2017) 6(7):67. 10.3390/jcm6070067 PMC5532575 28698517

[21] Basdeo SA Cluxton D Sulaimani J Moran B Canavan M Orr C Ex-Th17 (Nonclassical Th1) Cells Are Functionally Distinct from Classical Th1 and Th17 Cells and Are Not Constrained by Regulatory T Cells. J Immunol (2017) 198(6):2249–59. 10.4049/jimmunol.1600737 28167631

[22] Ramstein J Broos CE Simpson LJ Ansel KM Sun SA Ho ME IFN-gamma-Producing T-Helper 17.1 Cells Are Increased in Sarcoidosis and Are More Prevalent than T-Helper Type 1 Cells. Am J Respir Crit Care Med (2016) 193(11):1281–91. 10.1164/rccm.201507-1499OC PMC4910899 26649486

[23] Broos CE Koth LL van Nimwegen M In ‘t Veen J Paulissen SMJ van Hamburg JP Increased T-helper 17.1 cells in sarcoidosis mediastinal lymph nodes. Eur Respir J (2018) 51(3). 10.1183/13993003.01124-2017 29449421

[24] Kaiser Y Lepzien R Kullberg S Eklund A Smed-Sorensen A Grunewald J Expanded lung T-bet+RORgammaT+ CD4+ T-cells in sarcoidosis patients with a favourable disease phenotype. Eur Respir J (2016) 48(2):484–94. 10.1183/13993003.00092-2016 27230441

[25] McAlees JW Lajoie S Dienger K Sproles AA Richgels PK Yang Y Differential control of CD4(+) T-cell subsets by the PD-1/PD-L1 axis in a mouse model of allergic asthma. Eur J Immunol (2015) 45(4):1019–29. 10.1002/eji.201444778 PMC4440042 25630305

[26] Suresh K Naidoo J Zhong Q Xiong Y Mammen J de Flores MV The alveolar immune cell landscape is dysregulated in checkpoint inhibitor pneumonitis. J Clin Invest (2019) 130:4305–15. 10.1172/JCI128654 PMC6763233 31310589

[27] Costello PJ Winchester RJ Curran SA Peterson KS Kane DJ Bresnihan B Psoriatic arthritis joint fluids are characterized by CD8 and CD4 T cell clonal expansions appear antigen driven. J Immunol (2001) 166(4):2878–86. 10.4049/jimmunol.166.4.2878 11160357

[28] Subudhi SK Aparicio A Gao J Zurita AJ Araujo JC Logothetis CJ Clonal expansion of CD8 T cells in the systemic circulation precedes development of ipilimumab-induced toxicities. Proc Natl Acad Sci U S A (2016) 113(42):11919–24. 10.1073/pnas.1611421113 PMC5081579 27698113

[29] Arakawa A Vollmer S Tietze J Galinski A Heppt MV Burdek M Clonality of CD4(+) Blood T Cells Predicts Longer Survival With CTLA4 or PD-1 Checkpoint Inhibition in Advanced Melanoma. Front Immunol (2019) 10:1336. 10.3389/fimmu.2019.01336 31275310 PMC6591437

[30] Johnson DB McDonnell WJ Gonzalez-Ericsson PI Al-Rohil RN Mobley BC Salem JE A case report of clonal EBV-like memory CD4(+) T cell activation in fatal checkpoint inhibitor-induced encephalitis. Nat Med (2019) 25(8):1243–50. 10.1038/s41591-019-0523-2 PMC6689251 31332390

[31] Oh DY Cham J Zhang L Fong G Kwek SS Klinger M Immune Toxicities Elicted by CTLA-4 Blockade in Cancer Patients Are Associated with Early Diversification of the T-cell Repertoire. Cancer Res (2017) 77(6):1322–30. 10.1158/0008-5472.CAN-16-2324 PMC5398199 28031229

[32] Stübig T Badbaran A Luetkens T Hildebrandt Y Atanackovic D Binder TMC 5-Azacytidine Promotes an Inhibitory T-Cell Phenotype and Impairs Immune Mediated Antileukemic Activity. Mediators Inflamm (2014) 2014:418292. 10.1155/2014/418292 24757283 PMC3976863

[33] Jia X Yang W Zhou X Han L Shi J Influence of demethylation on regulatory T and Th17 cells in myelodysplastic syndrome. Oncol Lett (2020) 19(1):442–8. 10.3892/ol.2019.11114 PMC6924080 31897157

[34] Wilson CB Rowell E Sekimata M Epigenetic control of T-helper-cell differentiation. Nat Rev Immunol (2009) 9(2):91–105. 10.1038/nri2487 19151746

