# Supplementary material for: Distinct Immunophenotypes of T Cells in Bronchoalveolar Lavage Fluid From Leukemia Patients With Immune Checkpoint Inhibitors-Related Pulmonary Complications
Source: Front Immunol. 2021 Jan 21;11:590494. doi: 10.3389/fimmu.2020.590494 (PMC7859512; doi:10.3389/fimmu.2020.590494)
Supplement: Supplementary file 1 [file Presentation_1.pptx]

## Slide 1
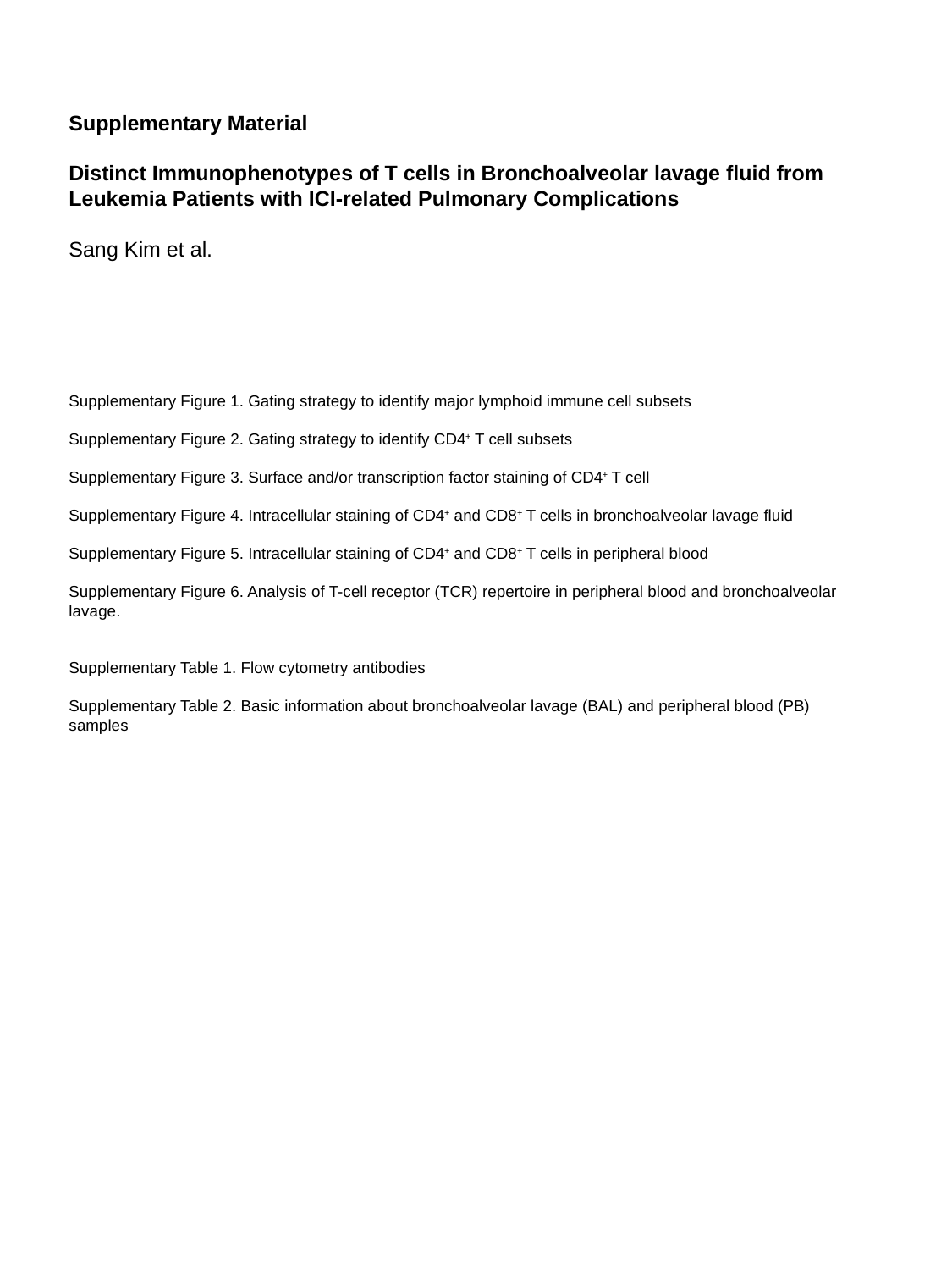

Supplementary Material
Distinct Immunophenotypes of T cells in Bronchoalveolar lavage fluid from Leukemia Patients with ICI-related Pulmonary Complications
Sang Kim et al.
Supplementary Figure 1. Gating strategy to identify major lymphoid immune cell subsets
Supplementary Figure 2. Gating strategy to identify CD4+ T cell subsets
Supplementary Figure 3. Surface and/or transcription factor staining of CD4+ T cell
Supplementary Figure 4. Intracellular staining of CD4+ and CD8+ T cells in bronchoalveolar lavage fluid
Supplementary Figure 5. Intracellular staining of CD4+ and CD8+ T cells in peripheral blood
Supplementary Figure 6. Analysis of T-cell receptor (TCR) repertoire in peripheral blood and bronchoalveolar lavage.
Supplementary Table 1. Flow cytometry antibodies
Supplementary Table 2. Basic information about bronchoalveolar lavage (BAL) and peripheral blood (PB) samples

## Slide 2
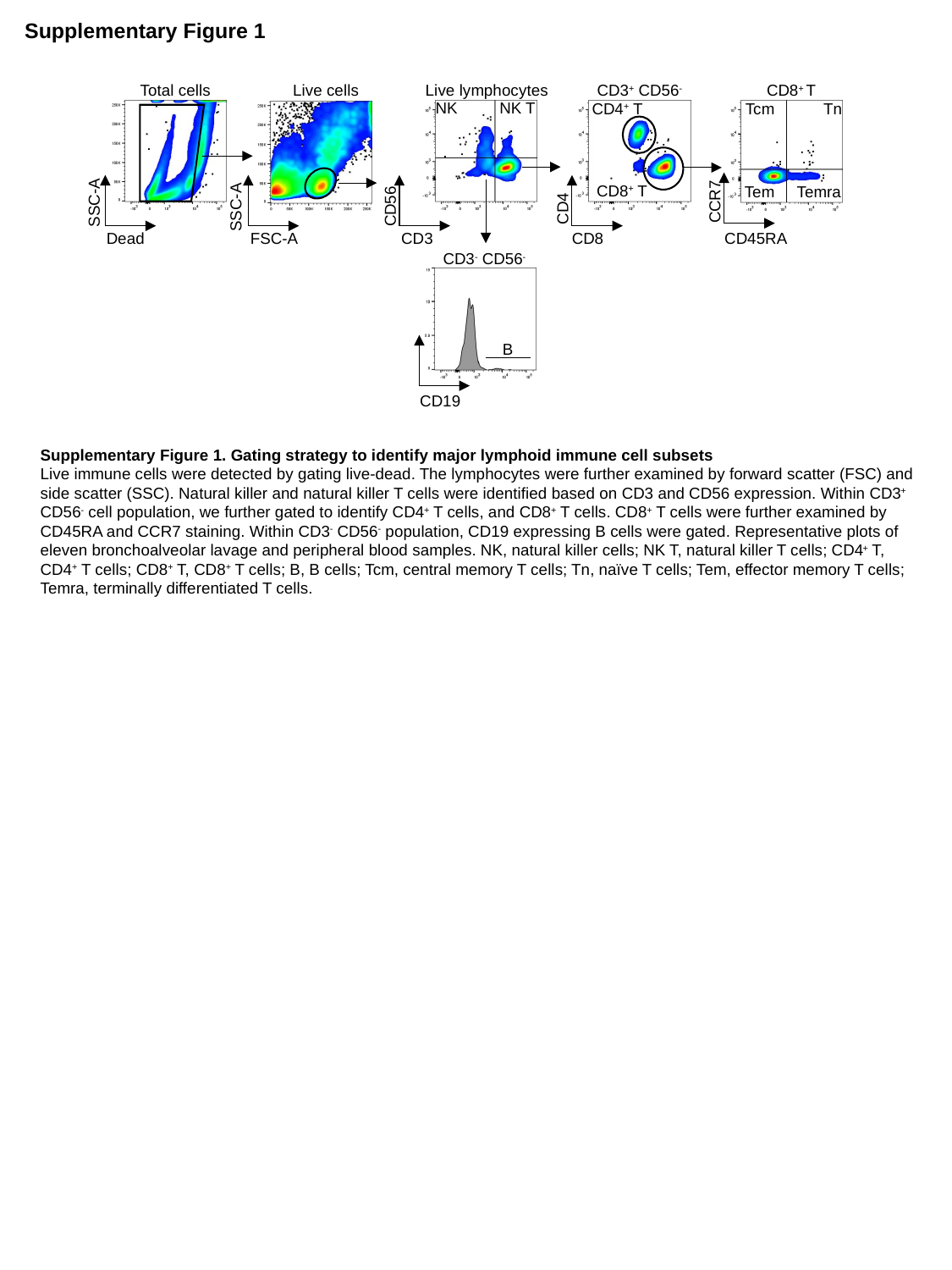

Supplementary Figure 1
Total cells
Live cells
Live lymphocytes
CD3+ CD56-
CD8+ T
CD4+ T
NK
NK T
Tcm
Tn
SSC-A
Dead
CCR7
CD45RA
SSC-A
FSC-A
CD56
CD3
CD8+ T
CD4
CD8
Tem
Temra
CD3- CD56-
CD19
B
Supplementary Figure 1. Gating strategy to identify major lymphoid immune cell subsets
Live immune cells were detected by gating live-dead. The lymphocytes were further examined by forward scatter (FSC) and side scatter (SSC). Natural killer and natural killer T cells were identified based on CD3 and CD56 expression. Within CD3+ CD56- cell population, we further gated to identify CD4+ T cells, and CD8+ T cells. CD8+ T cells were further examined by CD45RA and CCR7 staining. Within CD3- CD56- population, CD19 expressing B cells were gated. Representative plots of eleven bronchoalveolar lavage and peripheral blood samples. NK, natural killer cells; NK T, natural killer T cells; CD4+ T, CD4+ T cells; CD8+ T, CD8+ T cells; B, B cells; Tcm, central memory T cells; Tn, naïve T cells; Tem, effector memory T cells; Temra, terminally differentiated T cells.

## Slide 3
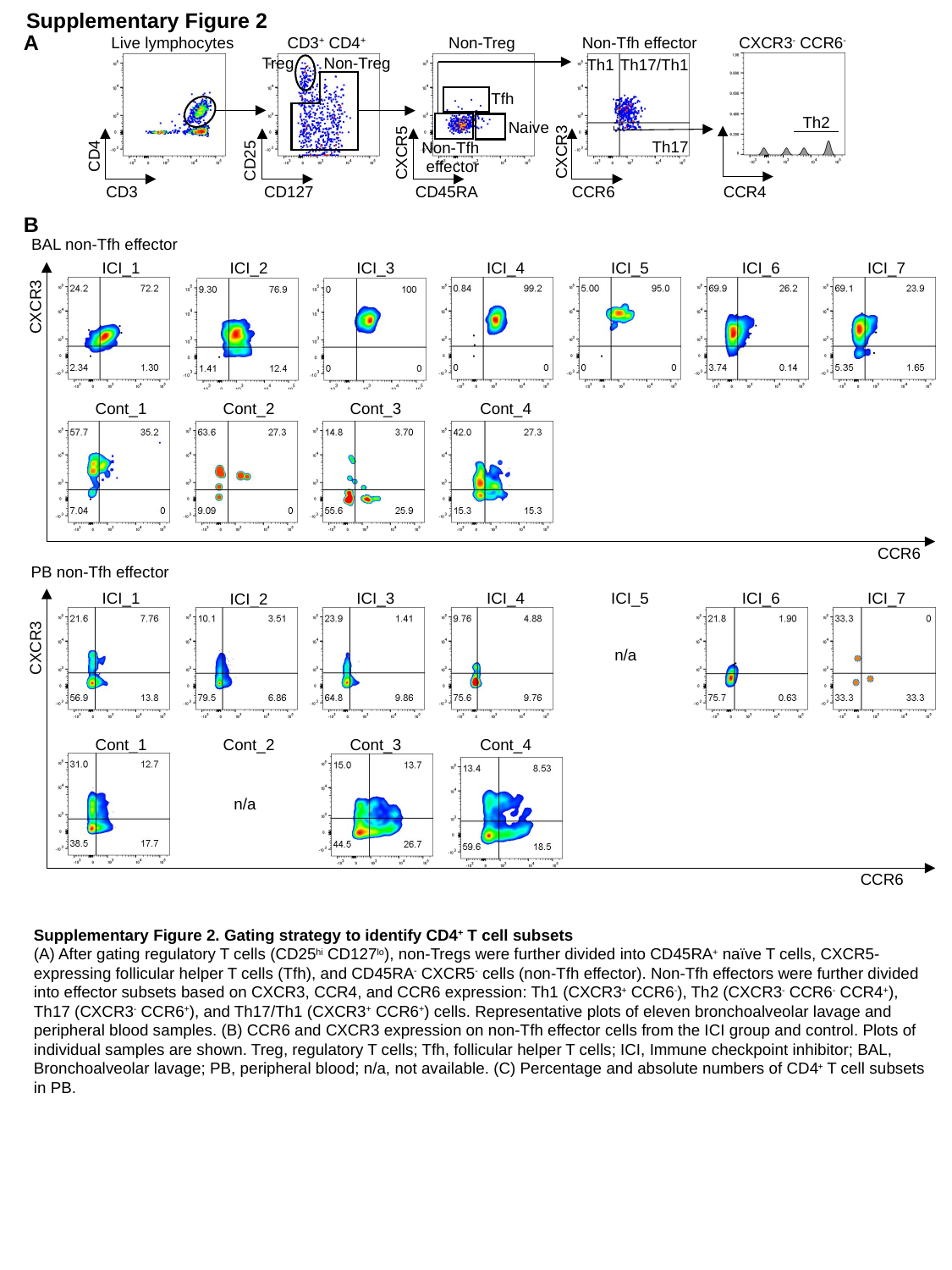

Supplementary Figure 2
A
Live lymphocytes
CD3+ CD4+
Non-Treg
Non-Tfh effector
CXCR3- CCR6-
Treg
Non-Treg
Th1
Th17/Th1
Tfh
CXCR3
CCR6
Th2
CXCR5
CD45RA
Naive
CCR4
CD4
CD3
CD25
CD127
Th17
Non-Tfh effector
B
BAL non-Tfh effector
ICI_1
ICI_2
ICI_3
ICI_4
ICI_5
ICI_6
ICI_7
CXCR3
Cont_1
Cont_2
Cont_3
Cont_4
CCR6
PB non-Tfh effector
ICI_1
ICI_3
ICI_4
ICI_5
ICI_6
ICI_7
ICI_2
CXCR3
n/a
Cont_1
Cont_2
Cont_3
Cont_4
n/a
CCR6
Supplementary Figure 2. Gating strategy to identify CD4+ T cell subsets
(A) After gating regulatory T cells (CD25hi CD127lo), non-Tregs were further divided into CD45RA+ naïve T cells, CXCR5-expressing follicular helper T cells (Tfh), and CD45RA- CXCR5- cells (non-Tfh effector). Non-Tfh effectors were further divided into effector subsets based on CXCR3, CCR4, and CCR6 expression: Th1 (CXCR3+ CCR6-), Th2 (CXCR3- CCR6- CCR4+), Th17 (CXCR3- CCR6+), and Th17/Th1 (CXCR3+ CCR6+) cells. Representative plots of eleven bronchoalveolar lavage and peripheral blood samples. (B) CCR6 and CXCR3 expression on non-Tfh effector cells from the ICI group and control. Plots of individual samples are shown. Treg, regulatory T cells; Tfh, follicular helper T cells; ICI, Immune checkpoint inhibitor; BAL, Bronchoalveolar lavage; PB, peripheral blood; n/a, not available. (C) Percentage and absolute numbers of CD4+ T cell subsets in PB.

## Slide 4
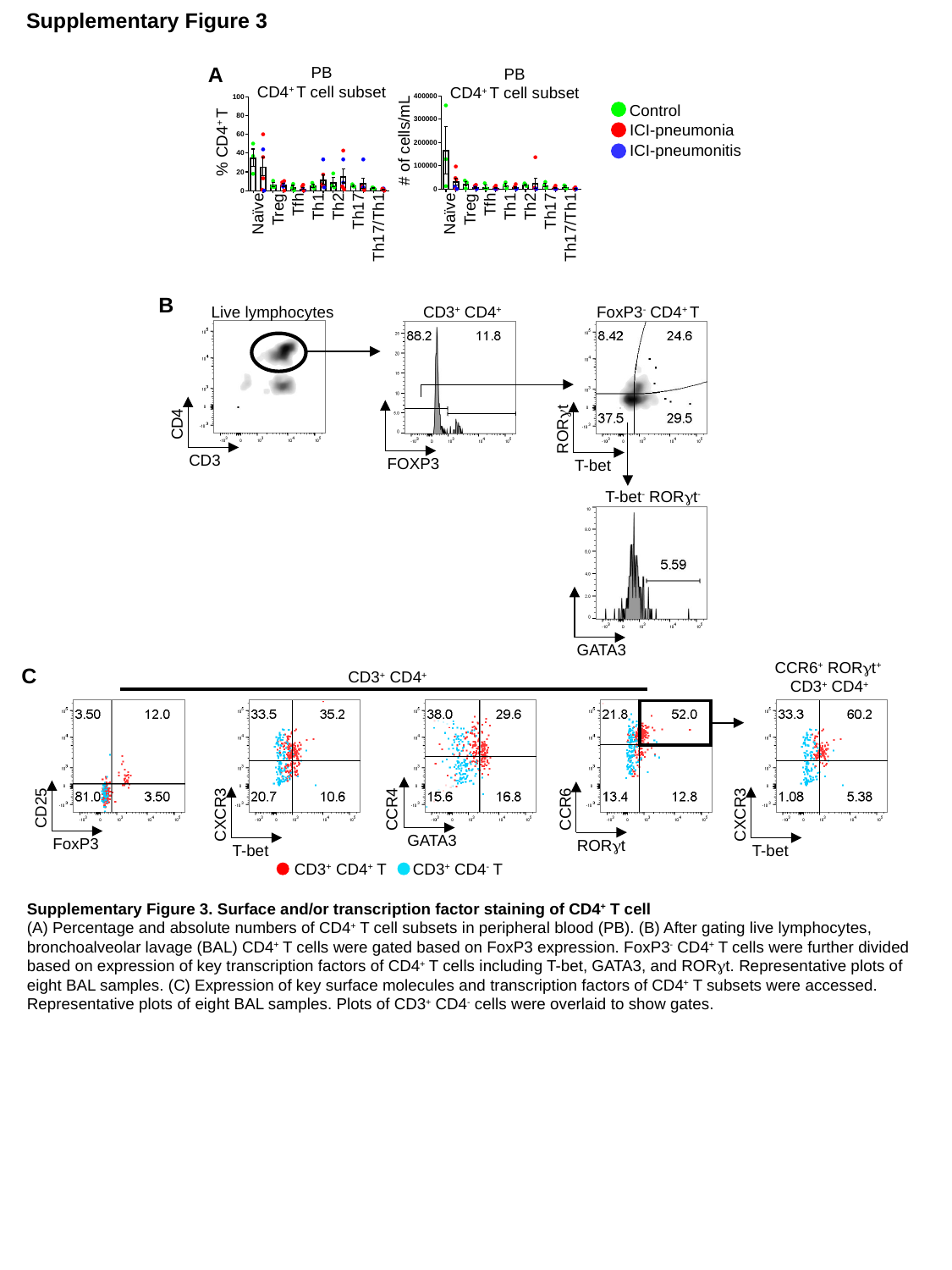

Supplementary Figure 3
A
PB
CD4+ T cell subset
PB
CD4+ T cell subset
Control
ICI-pneumonia
ICI-pneumonitis
% CD4+ T
# of cells/mL
Tfh
Th1
Th2
Treg
Th17
Naïve
Th17/Th1
Tfh
Th1
Th2
Treg
Th17
Naïve
Th17/Th1
B
Live lymphocytes
CD3+ CD4+
FoxP3- CD4+ T
RORgt
T-bet
CD4
CD3
FOXP3
T-bet- RORgt-
GATA3
CCR6+ RORgt+
CD3+ CD4+
C
CD3+ CD4+
CD25
FoxP3
CXCR3
T-bet
CCR4
GATA3
CCR6
RORgt
CXCR3
T-bet
CD3+ CD4+ T CD3+ CD4- T
Supplementary Figure 3. Surface and/or transcription factor staining of CD4+ T cell
(A) Percentage and absolute numbers of CD4+ T cell subsets in peripheral blood (PB). (B) After gating live lymphocytes, bronchoalveolar lavage (BAL) CD4+ T cells were gated based on FoxP3 expression. FoxP3- CD4+ T cells were further divided based on expression of key transcription factors of CD4+ T cells including T-bet, GATA3, and RORgt. Representative plots of eight BAL samples. (C) Expression of key surface molecules and transcription factors of CD4+ T subsets were accessed. Representative plots of eight BAL samples. Plots of CD3+ CD4- cells were overlaid to show gates.

## Slide 5
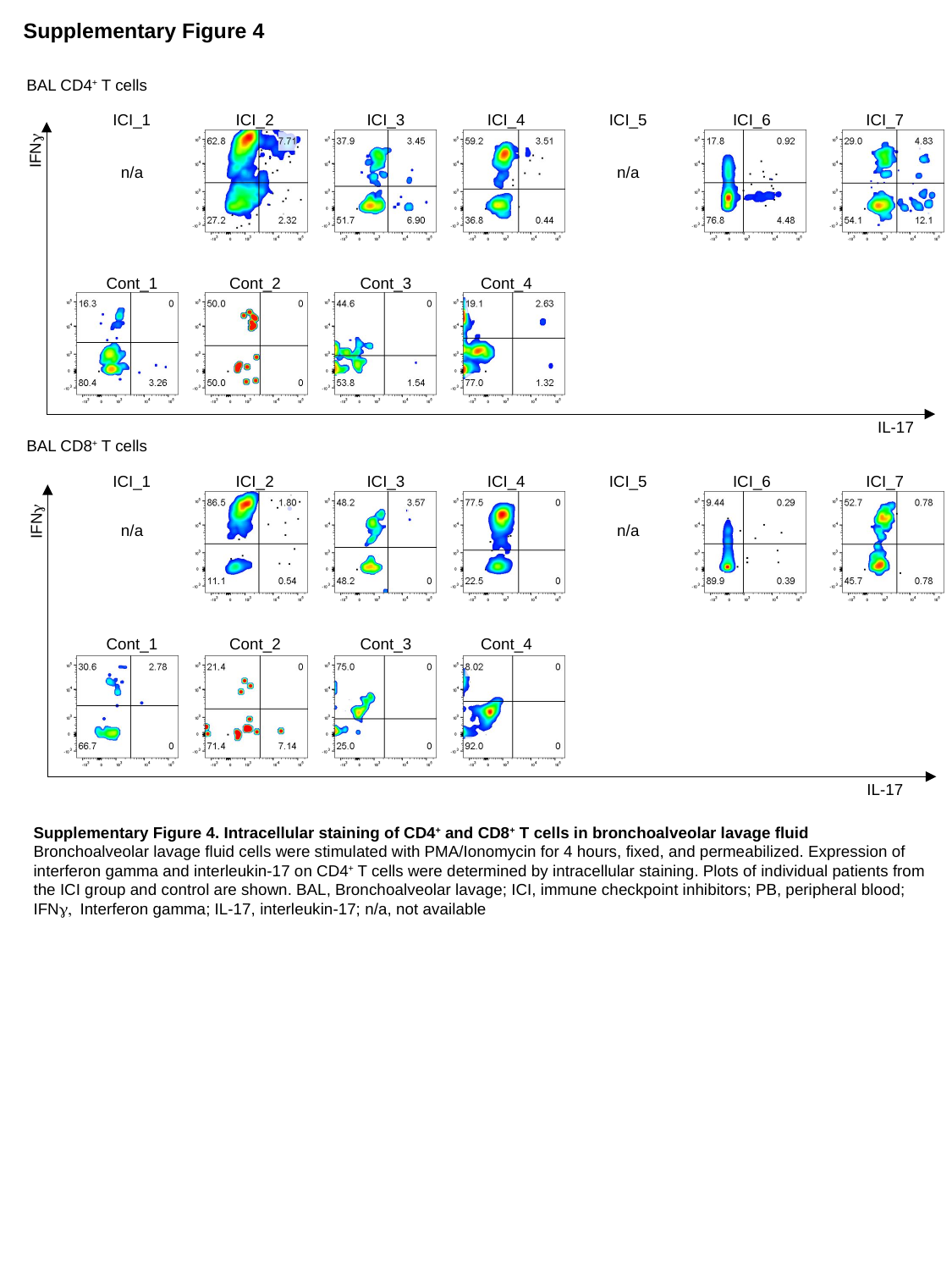

Supplementary Figure 4
BAL CD4+ T cells
ICI_1
ICI_2
ICI_3
ICI_4
ICI_5
ICI_6
ICI_7
IFNg
IL-17
n/a
n/a
Cont_1
Cont_2
Cont_3
Cont_4
BAL CD8+ T cells
ICI_1
ICI_2
ICI_3
ICI_4
ICI_5
ICI_6
ICI_7
IFNg
IL-17
n/a
n/a
Cont_1
Cont_2
Cont_3
Cont_4
Supplementary Figure 4. Intracellular staining of CD4+ and CD8+ T cells in bronchoalveolar lavage fluid
Bronchoalveolar lavage fluid cells were stimulated with PMA/Ionomycin for 4 hours, fixed, and permeabilized. Expression of interferon gamma and interleukin-17 on CD4+ T cells were determined by intracellular staining. Plots of individual patients from the ICI group and control are shown. BAL, Bronchoalveolar lavage; ICI, immune checkpoint inhibitors; PB, peripheral blood; IFNg, Interferon gamma; IL-17, interleukin-17; n/a, not available

## Slide 6
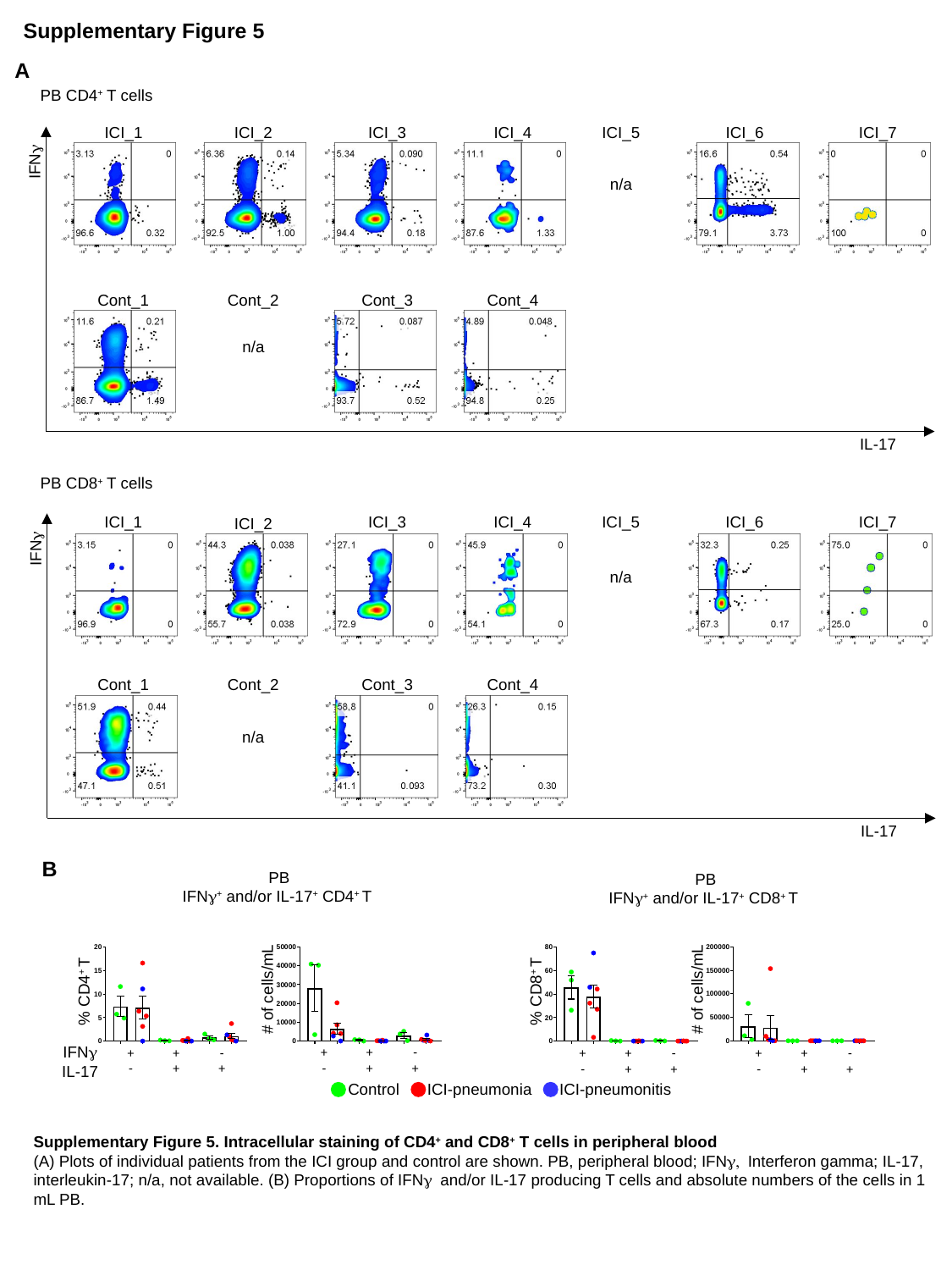

Supplementary Figure 5
A
PB CD4+ T cells
ICI_1
ICI_2
ICI_3
ICI_4
ICI_5
ICI_6
ICI_7
IFNg
IL-17
n/a
Cont_1
Cont_2
Cont_3
Cont_4
n/a
PB CD8+ T cells
ICI_1
ICI_3
ICI_4
ICI_5
ICI_6
ICI_7
ICI_2
IFNg
IL-17
n/a
Cont_1
Cont_2
Cont_3
Cont_4
n/a
B
PB
IFNg+ and/or IL-17+ CD4+ T
PB
IFNg+ and/or IL-17+ CD8+ T
% CD4+ T
# of cells/mL
% CD8+ T
# of cells/mL
IFNg
IL-17
+
-
+
+
-
+
+
-
+
+
-
+
+
-
+
+
-
+
+
-
+
+
-
+
Control
ICI-pneumonia
ICI-pneumonitis
Supplementary Figure 5. Intracellular staining of CD4+ and CD8+ T cells in peripheral blood
(A) Plots of individual patients from the ICI group and control are shown. PB, peripheral blood; IFNg, Interferon gamma; IL-17, interleukin-17; n/a, not available. (B) Proportions of IFNg and/or IL-17 producing T cells and absolute numbers of the cells in 1 mL PB.

## Slide 7
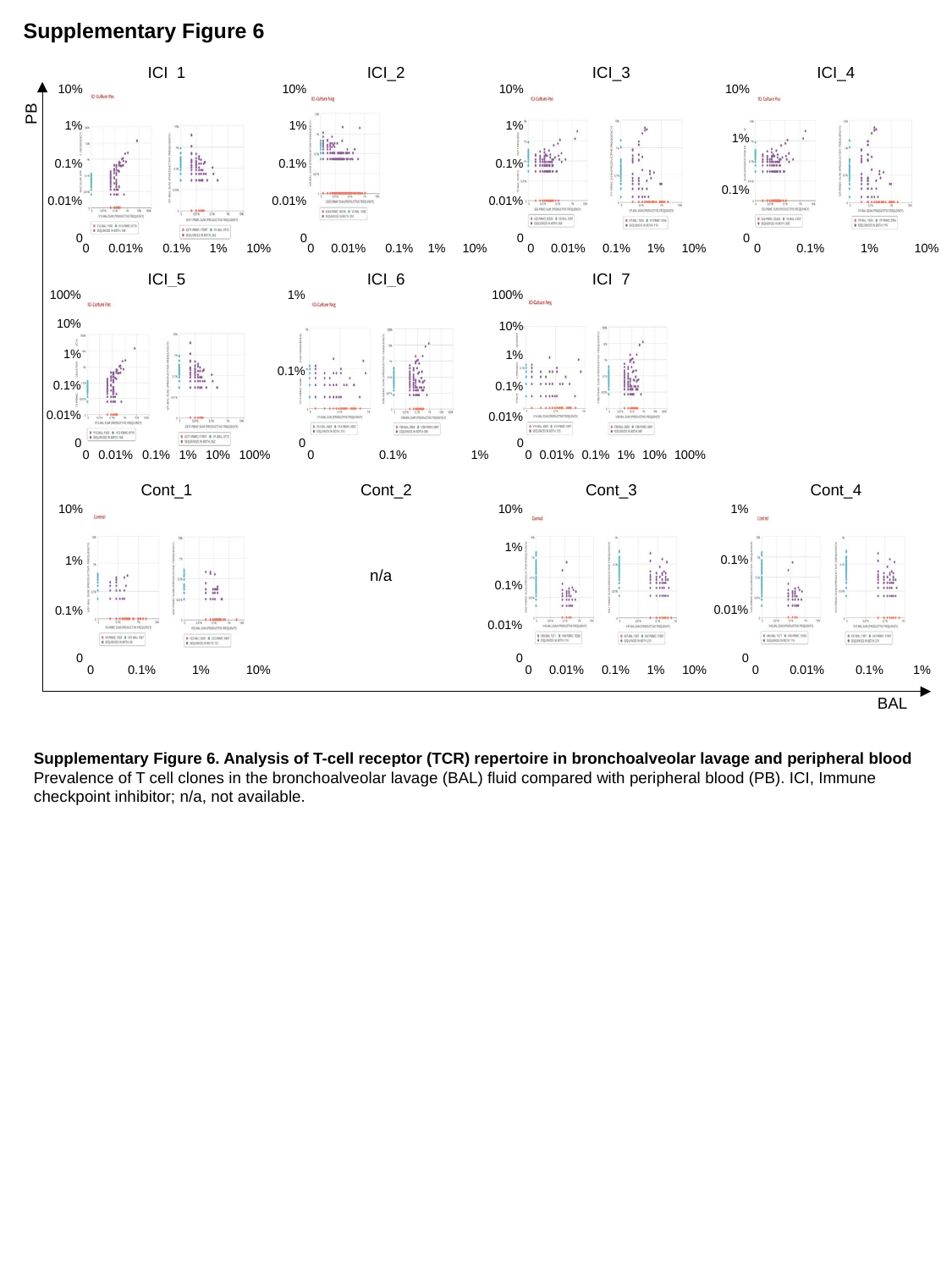

Supplementary Figure 6
ICI_1
ICI_2
ICI_3
ICI_4
10%
10%
10%
10%
PB
1%
1%
1%
1%
0.1%
0.1%
0.1%
0.1%
0.01%
0.01%
0.01%
0
0
0
0
0
0.01%
0.1%
1%
10%
0
0.01%
0.1%
1%
10%
0
0.01%
0.1%
1%
10%
0
0.1%
1%
10%
ICI_5
ICI_6
ICI_7
100%
1%
100%
10%
10%
1%
1%
0.1%
0.1%
0.1%
0.01%
0.01%
0
0
0
0
0.01%
0.1%
1%
10%
100%
0
0.1%
1%
0
0.01%
0.1%
1%
10%
100%
Cont_1
Cont_2
Cont_3
Cont_4
10%
10%
1%
1%
0.1%
1%
n/a
0.1%
0.01%
0.1%
0.01%
0
0
0
0
0.1%
1%
10%
0
0.01%
0.1%
1%
10%
0
0.01%
0.1%
1%
BAL
Supplementary Figure 6. Analysis of T-cell receptor (TCR) repertoire in bronchoalveolar lavage and peripheral blood
Prevalence of T cell clones in the bronchoalveolar lavage (BAL) fluid compared with peripheral blood (PB). ICI, Immune checkpoint inhibitor; n/a, not available.

## Slide 8
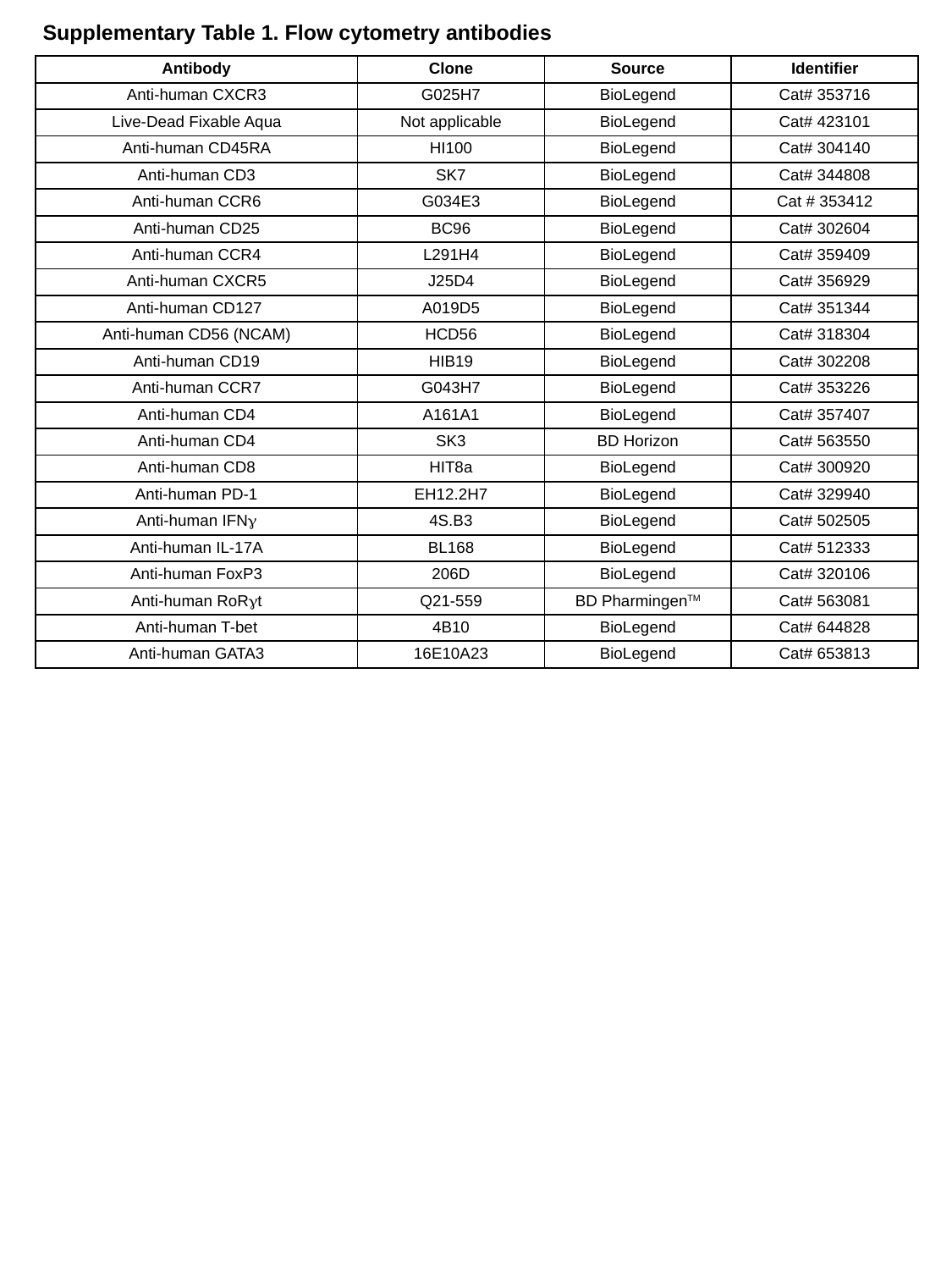

Supplementary Table 1. Flow cytometry antibodies
| Antibody | Clone | Source | Identifier |
| --- | --- | --- | --- |
| Anti-human CXCR3 | G025H7 | BioLegend | Cat# 353716 |
| Live-Dead Fixable Aqua | Not applicable | BioLegend | Cat# 423101 |
| Anti-human CD45RA | HI100 | BioLegend | Cat# 304140 |
| Anti-human CD3 | SK7 | BioLegend | Cat# 344808 |
| Anti-human CCR6 | G034E3 | BioLegend | Cat # 353412 |
| Anti-human CD25 | BC96 | BioLegend | Cat# 302604 |
| Anti-human CCR4 | L291H4 | BioLegend | Cat# 359409 |
| Anti-human CXCR5 | J25D4 | BioLegend | Cat# 356929 |
| Anti-human CD127 | A019D5 | BioLegend | Cat# 351344 |
| Anti-human CD56 (NCAM) | HCD56 | BioLegend | Cat# 318304 |
| Anti-human CD19 | HIB19 | BioLegend | Cat# 302208 |
| Anti-human CCR7 | G043H7 | BioLegend | Cat# 353226 |
| Anti-human CD4 | A161A1 | BioLegend | Cat# 357407 |
| Anti-human CD4 | SK3 | BD Horizon | Cat# 563550 |
| Anti-human CD8 | HIT8a | BioLegend | Cat# 300920 |
| Anti-human PD-1 | EH12.2H7 | BioLegend | Cat# 329940 |
| Anti-human IFNg | 4S.B3 | BioLegend | Cat# 502505 |
| Anti-human IL-17A | BL168 | BioLegend | Cat# 512333 |
| Anti-human FoxP3 | 206D | BioLegend | Cat# 320106 |
| Anti-human RoRgt | Q21-559 | BD PharmingenTM | Cat# 563081 |
| Anti-human T-bet | 4B10 | BioLegend | Cat# 644828 |
| Anti-human GATA3 | 16E10A23 | BioLegend | Cat# 653813 |

## Slide 9
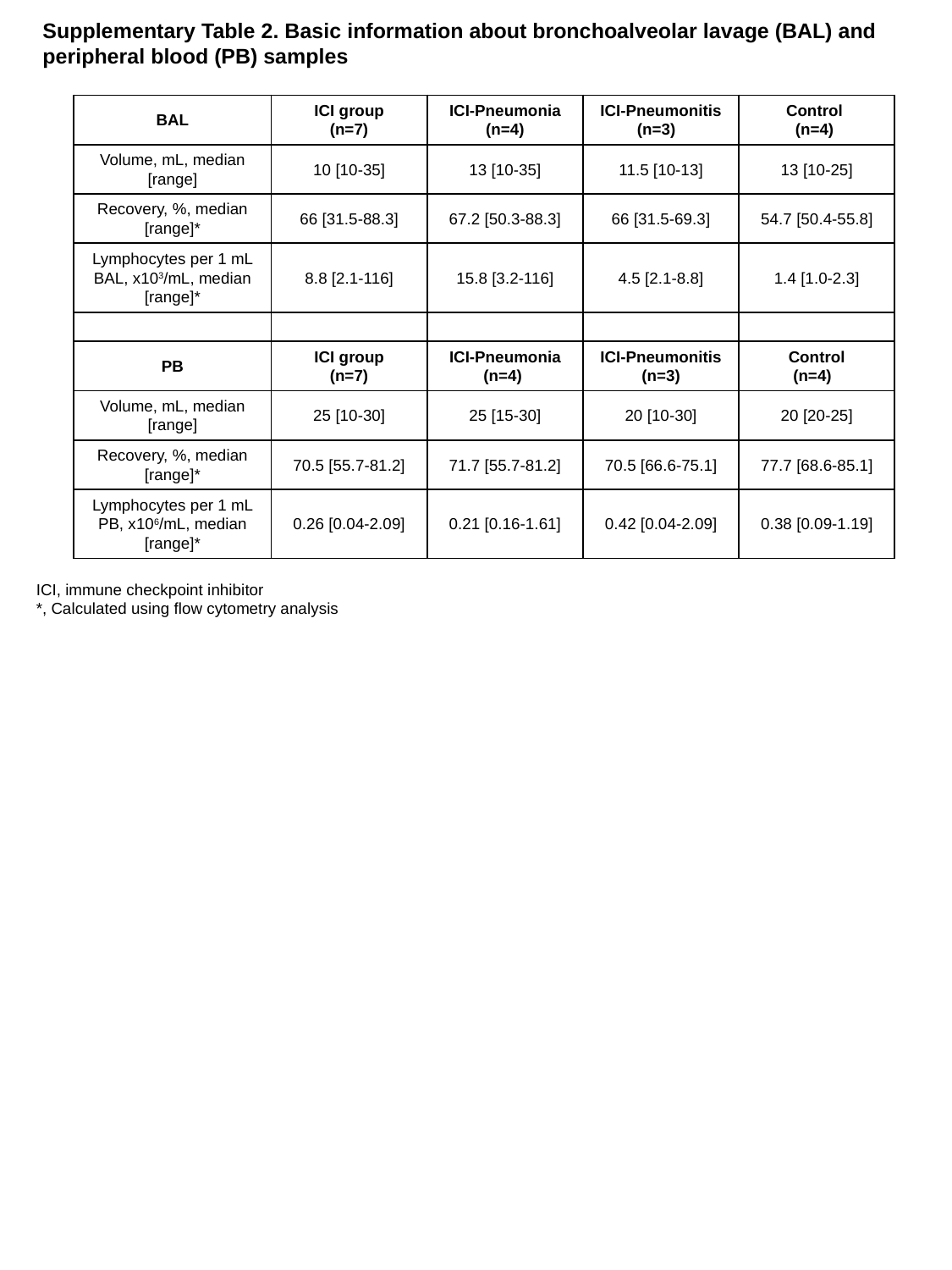

Supplementary Table 2. Basic information about bronchoalveolar lavage (BAL) and peripheral blood (PB) samples
| BAL | ICI group (n=7) | ICI-Pneumonia (n=4) | ICI-Pneumonitis (n=3) | Control (n=4) |
| --- | --- | --- | --- | --- |
| Volume, mL, median [range] | 10 [10-35] | 13 [10-35] | 11.5 [10-13] | 13 [10-25] |
| Recovery, %, median [range]\* | 66 [31.5-88.3] | 67.2 [50.3-88.3] | 66 [31.5-69.3] | 54.7 [50.4-55.8] |
| Lymphocytes per 1 mL BAL, x103/mL, median [range]\* | 8.8 [2.1-116] | 15.8 [3.2-116] | 4.5 [2.1-8.8] | 1.4 [1.0-2.3] |
| | | | | |
| PB | ICI group (n=7) | ICI-Pneumonia (n=4) | ICI-Pneumonitis (n=3) | Control (n=4) |
| Volume, mL, median [range] | 25 [10-30] | 25 [15-30] | 20 [10-30] | 20 [20-25] |
| Recovery, %, median [range]\* | 70.5 [55.7-81.2] | 71.7 [55.7-81.2] | 70.5 [66.6-75.1] | 77.7 [68.6-85.1] |
| Lymphocytes per 1 mL PB, x106/mL, median [range]\* | 0.26 [0.04-2.09] | 0.21 [0.16-1.61] | 0.42 [0.04-2.09] | 0.38 [0.09-1.19] |
ICI, immune checkpoint inhibitor
*, Calculated using flow cytometry analysis
